# Rotation of Multiple Single-Gene Transgenic Crops Did Not Slow the Evolution of Resistance to Cry1F or Cry1Ie in *Ostrinia furnacalis*

**DOI:** 10.3390/insects14010074

**Published:** 2023-01-12

**Authors:** Yueqin Wang, Yudong Quan, Zhenying Wang, Kanglai He

**Affiliations:** State Key Laboratory for Biology of Plant Diseases and Insect Pests, Institute of Plant Protection, Chinese Academy of Agricultural Sciences, Beijing 100193, China

**Keywords:** *Bacillus thuringiensis*, resistance, rotation, *Ostrinia furnacalis*, resistance management

## Abstract

**Simple Summary:**

Delaying or preventing the evolution of resistance to *Bacillus thuringiensis* (Bt) toxins produced by transgenic crops in insect pests is a major challenge for agriculture. A simulation model suggested that the rotation of different single-gene crops planted in subsequent seasons has the potential to delay resistance. To test this prediction, we set out laboratory selection experiments under the alternation of multi-toxins by mixing individual toxins (Cry1Ab, Cry1F, and Cry1Ie) in an artificial diet to emulate single-gene Bt maize plants. Two (Cry1Ab-Cry1F, Cry1Ab-Cry1Ie) or three (Cry1Ab–Cry1F–Cry1Ie) toxin alternation regimes were tested to imitate two or three single-gene crops in a rotation fashion. The species tested was the Asian corn borer, *Ostrinia furnacalis* (Guenée), the most economically important species of maize pest in Asia. The present study suggested that rotation of multiple toxins did not slow the evolution of resistance to Cry1F or Cry1Ie. Data generated from the study will assist in the development of sustainable resistance-management strategies.

**Abstract:**

A common strategy for delaying the evolution of resistance to transgenic crops that produce insecticidal proteins from *Bacillus thuringiensis* is to ensure that insect pests are exposed to multiple toxins with different mechanisms of action (MoAs). This can take the form of planting crops in a rotation pattern when different crops expressing single toxins are available on the market. The efficacy of a rotation strategy is reliant on mathematical models based on biological assumptions. Here, we designed laboratory evolution experiments to test whether Bt-based insecticidal proteins with different MoAs used in rotation could delay resistance from developing in Asian corn borer (ACB), *Ostrinia furnacalis*. We investigated the proteins Cry1Ab, Cry1F, and Cry1Ie, which are widely utilized for commercial insect control. We found that rotation of multiple toxins did not slow the evolution of resistance to Cry1F or Cry1Ie. Furthermore, the evolution of ACB to the Cry1Ab toxin develops faster when Cry1F or Cry1Ie is present, as compared to Cry1Ab exposure only. Our results suggest that toxins used in a rotation fashion do not work as an effective strategy in delaying ACB resistance evolution to Cry toxins over one-toxin exposure. Our result highlights the need to better understand the biological factors leading to insecticidal protein resistance and to develop IRM strategies against target insects.

## 1. Introduction

*Bacillus thuringiensis* (Bt)-derived crystal proteins, so-called “Cry toxins”, have had over 50 years of safe use as foliar insecticides [1,2]. In the last two decades, biotechnology has allowed for the expression of these proteins directly in crop plants [3]. These transgenic crops express the proteins in planta and, thereby have led to some agronomic, environmental, and economic benefits compared to conventional crops [4,5].

A variety of target insects have developed resistance to Bt transgenic crops in the field [6,7]. Both agribusinesses and regulatory agencies would like to prevent or delay the evolution of resistance in targeted pests to insecticidal Cry toxins. Strategies, such as high-dose toxin expression, refuge areas, pyramids, mosaic, and rotation, have been employed in an attempt to delay resistance [8,9,10,11,12]. However, the problem is that these strategies are often based largely on assumptions. For example, under ideal conditions, pyramided crops that produce two or more distinct toxins that kill the same pest can substantially slow the evolution of pest resistance [12]. However, unlike the nearly ideal conditions of the model system, a review of the relevant data indicates that, in many cases, each toxin in a pyramid does not meet high doses, resistance is not recessive, and some cross-resistance occurs between the toxins in these pyramids [13,14]. Furthermore, refuge abundance is often limited. Our previous study concluded that evolution of resistance by Asian corn borer (ACB), *Ostrinia furnacalis*, was not consistently delayed by pairs of Bt toxins relative to the same toxins used singly under nearly worst-case conditions [15].

A strategy somewhat similar to pyramiding is “toxin rotation” where two or more different crops that express different toxins are planted in subsequent seasons across generations, or different insecticides of different classes are applied across generations of the target pests [16,17]. The surviving resistant alleles from the first crop season should be eliminated by a different toxin during the next rotation (season). Theoretically, such a practice could reduce the evolution of resistance and it does not require a high dose. However, the rotation strategy makes the assumption that a single or linked allele will not convey resistance to both toxins, inheritance of resistance will be recessive, and fitness disadvantages will be present [16]. If a given individual has an allele for resistance to one of the two crops, it will be killed. If a different insect has a different allele conferring resistance to the other event, it too will be killed [12]. It further supposes that selected individuals will stay in a similar geographic area, which is a large assumption given that larvae surviving to the adult stage can fly to other fields [17]. However, in spite of modeling efforts, experimental data from selection experiments testing toxins, singly and in alternation, are limited [16]. Moreover, unlike the nearly ideal conditions of the model system, a review of the relevant data indicates that, in many cases, a toxin (or insecticide) in alternation does not kill nearly all susceptible targets and some cross-resistance occurs.

Given these uncertainties, we set out laboratory selection experiments to examine the evolutionary resistance trend in *O. furnacalis*, an allied species of the European corn borer (ECB, *O. nubilalis*) and the most economically important species of maize pests in Asia [18]. A few widely planted single-gene crops of Bt maize expressing Cry1Ab or Cry1F toxins have been or were initially deployed to protect against this pest in Asian countries, including the Philippines and Vietnam [3]. Field trails have also been carried out, with those events as well as other events expressing the Cry1Ie toxin in China [19]. Hereby, resistance evolution experiments were conducted under the alternation of multi-toxins by mixing individual toxins (Cry1Ab, Cry1F, and Cry1Ie) in an artificial diet to emulate single-gene Bt maize plants. Two (Cry1Ab-Cry1F, Cry1Ab-Cry1Ie) or three (Cry1Ab-Cry1F-Cry1Ie-Cry1Ab) toxin alternation regimes were tested to imitate two or three single-gene crops in a rotation fashion. Diverse mechanisms of resistance to Bt toxins were identified, including reduced activation of protoxin to toxin, elevated immune response, faster regeneration of midgut cells, and toxin sequestration [2,20]. The most common one involves changes in receptor proteins that reduce the binding to larval midguts [21]. Previous binding assays demonstrated that Cry1Ie did not recognize the Cry1Ab or Cry1F binding sites in *O. nubilalis* brush border membrane vesicles [22], and laboratory selection with Cry1Ie caused no cross-resistance to Cry1Ab or Cry1F in *O. furnacalis* [23]. Asymmetrical cross-resistance has been reported between Cry1Ab and Cry1F in *O. furnacalis* [24,25]. A CRISPR/Cas9-mediated genome-editing study demonstrated that ABCC2 protein is a functional receptor for Cry1F but not for Cry1Ab in *O. furnacalis* [26]. All the evidence supported that the three Cry1 toxins may have their own target sites.

## 2. Materials and Methods

### 2.1. Source of O. furnacalis

A susceptible strain (*Of-S*) of the ACB was used as the original insect source for this study. *Of-S* was established from a population (560 pupae developed from diapause larvae) collected from a maize field near Yangling in western Shaanxi Province in 2012. In the last two decades, Bt products have not been used in this area. Larvae of *Of-S* were reared on an artificial diet at 27 ± 1 °C with 70–80% relative humidity (RH) and a photoperiod of 16:8 h (L:D). Standard ACB-rearing techniques were followed whilst insects were maintained in the laboratory [27].

### 2.2. Cry Toxins

Cry1Ab and Cry1F toxins (98% purity, trypsin-activated) that were commercial products produced by Marianne P. Carey at the Case Western Reserve University, Cleveland, OH, USA, were used in this study. Purified Cry1Ie toxin (>92% purity) expressed in recombinant *E. coli* [28] was obtained from ABZYMO Biosciences, Beijing, China.

### 2.3. Laboratory Selection Regimes

Resistance evolution experiments were performed by rearing larvae on artificial diet mixed with individual Bt toxins. The experiments included single-toxin continual selection (Cry1Ab, Cry1F, or Cry1Ie), two toxins in alternation (Cry1Ab-Cry1F, Cry1Ab-Cry1Ie), and three toxins in alternation (Cry1Ab-Cry1F-Cry1Ie-Cry1Ab, Cry1Ab-Cry1F-Cry1Ie-Cry1F-Cry1Ab). For each selection experiment, two containers with >10,000 neonates per container were initially set out. All pupae harvested from the two rearing containers were pooled and transferred into an oviposition screen cage. Offspring egg masses were used for bioassays and to carry on next generation of selection, respectively. All experiments were conducted for 14 generations of selection. Selection pressure was set up constantly across generations at a concentration of LC_50_ values for the *Of-S* strain derived from a seven-day bioassay, i.e., 0.2, 0.5, and 2.0 μg/g for Cry1Ab, Cry1F, and Cry1Ie, respectively [15]. This resulted in the establishment of seven distinct strains, i.e., individual toxin Cry1Ab-, Cry1F-, and Cry1Ie-selected strains *Of*-AbR, *Of*-FR, *Of*-IeR, two-toxin alternating selected strains, Bi-alt.1 and Bi-alt.2, and three-toxin alternating selected strains, Tri-alt.1 and Tri-alt.2.

### 2.4. Susceptibility Bioassays

Susceptibility of neonates to Cry1Ab, Cry1F, and Cry1Ie toxins was determined individually by diet-incorporation bioassays. The freshly prepared artificial diet was dispensed into wells in 48-well trays, which were then infested with 1 neonate (<12 h after hatching) per well and placed at 27 ± 1 °C and 70–80% relative humidity with a 16L: 8D h photoperiod. Mortality was recorded in 7 days and larvae that had not developed beyond first instar and weighed ≤ 0.1 mg were considered to be dead [15]. Each bioassay concentration was replicated twice, with 96 larvae tested in total.

For all the strains, we used bioassays with a range of 6 to 9 concentrations of Cry1Ab to evaluate susceptibility for the generation before selection (0) and each of the next 14 generations. For the strains selected with Cry1F or Cry1Ie, singly or in alternation with other toxin(s), we used the same approach to evaluate susceptibility to these toxins for generations 0 to 5. However, high levels of resistance to Cry1F and Cry1Ie evolved rapidly. To reduce the expense associated with the large amount of Cry1F and Cry1Ie toxins needed to kill larvae, we tested fewer generations with fewer concentrations of these two toxins from generations 6 to 14. 

### 2.5. Statistical Analysis

Concentration-mortality data were analyzed with a probit model using the PoloPlus program to yield LC_50_ values and 95% fiducial limits (FLs) for each generation separately. To assess and evaluate the development of resistance in a selected strain, the resistance ratio (RR) was calculated with a value of LC_50_ of a toxin for this strain divided by the value of LC_50_ of the same toxin for the susceptible strain *Of-S*. The 95% confidence intervals (CIs) for each RR were calculated using PoloPlus, which was then used to test if difference was statistically significant. We used the conservative criteria of no overlap between 95% FL for LC_50_ values and no overlap between 95% CIs and 1 for RRs. 

The Cry1Ab resistance ratio (RR) was tested in each generation for 14 generations. The Cry1Ie- and Cry1F-resistance rations were determined in each generation for the first 5 generations, then every 2 to 4 generations for the next 9 generations.

## 3. Results

**Figure 1:**
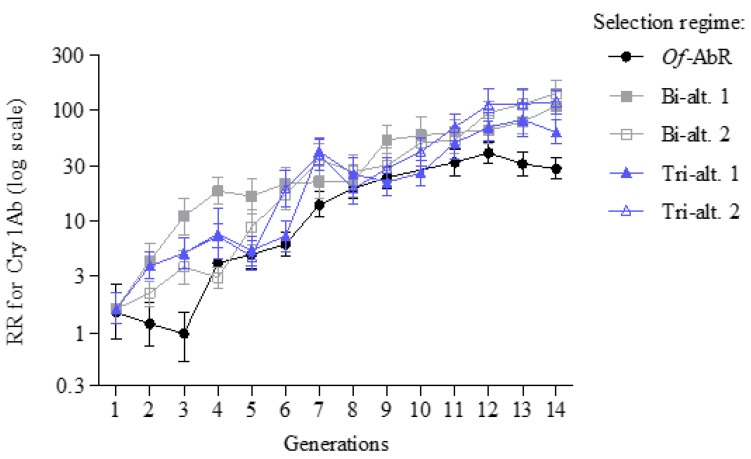
Evolution of resistance to Cry1Ab in *O. furnacalis* with different Bt toxin treatments. *Of*-AbR, selected with Cry1Ab alone. Bi-alt.1, selected with Cry1Ab-Cry1F in alternation. Bi-alt.2, selected with Cry1Ab-Cry1Ie in alternation. Tri-alt.1, selected with Cry1Ab-Cry1F-Cry1Ie in alternation. Tri-alt.2, selected with Cry1Ab-Cry1F-Cry1Ie-Cry1F in alternation.

We compared how quickly an insect population evolves resistance to each toxin when exposed to single or multiple toxins. After 14 generations, the RR for Cry1Ab was 28 (95% CI: 23–35), 104 (78–139), 135 (102–178), 60 (47–77), and 114 (89–146) for *Of*-AbR, Bi-alt.1, Bi-alt.2, Tri-alt.1, and Tri-alt.2, respectively. Based on the criterion of non-overlap of 95% CIs, the RR for Cry1Ab was significantly higher for Tri-alt.1 than *Of*-AbR, but it was significantly lower than other strains (Figure 1 and Table S1).

**Figure 2:**
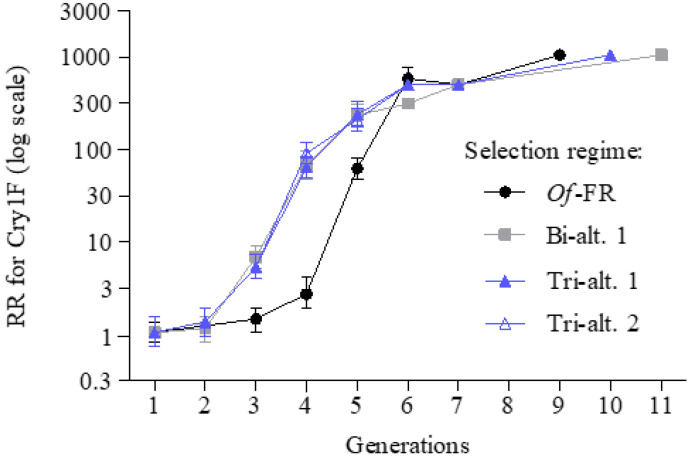
Evolution of resistance to Cry1F in *O. furnacalis* with different Bt toxin treatments. *Of*-FR, selected with Cry1F alone. Bi-alt.1, selected with Cry1Ab-Cry1F in alternation. Tri-alt.1, selected with Cry1Ab-Cry1F-Cry1Ie in alternation. Tri-alt.2, selected with Cry1Ab-Cry1F-Cry1Ie-Cry1F in alternation.

When experiments mimicking rotation with Cry1Ab and Cry1F were performed, resistance was able to evolve drastically towards Cry1F. Over the course of 14 generations, the resistance level to Cry1F was so high that the tested highest concentrations failed to kill 50% of the larvae and we could not accurately estimate the LC_50_ values and RRs. At generation 5, the RRs for Cry1F were 59 (45–78), 223 (171–291), 227 (165–313), and 200 (149–267) for *Of*-FR, Bi-alt.1 Tri-alt.1, and Tri-alt.2 (Figure 2 and Table S2).

**Figure 3:**
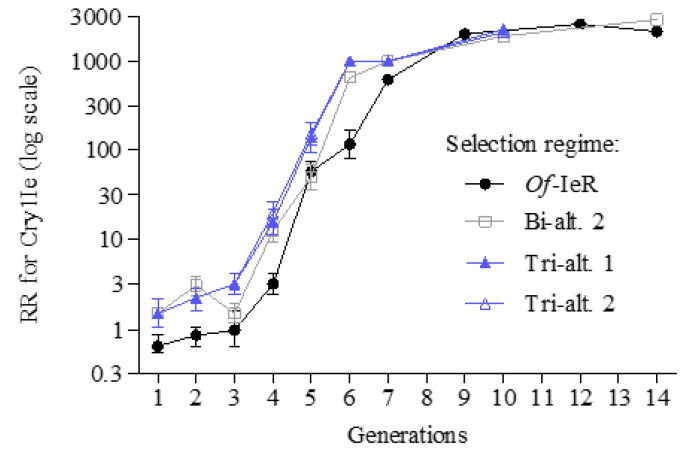
Evolution of resistance to Cry1Ie in *O. furnacalis* with different Bt toxin treatments. *Of*-IeR, selected with Cry1Ie alone. Bi-alt.2, selected with Cry1Ab-Cry1Ie in alternation. Tri-alt.1, selected with Cry1Ab-Cry1F-Cry1Ie in alternation. Tri-alt.2, selected with Cry1Ab-Cry1F-Cry1Ie-Cry1F in alternation.

Similar results were observed when Cry1Ie was in alternation-mimicking scheme: after five generations, the insects showed tolerance to Cry1Ie but not to Cry1Ab. For example, the RR for Cry1Ie did not differ between *Of*-IeR (55 (42–72)) and Bi-alt.2 (48 (34–67)), but both significantly lower than the other two treatments. The RRs were 133 (91–196) and 146 (110–193), respectively (Figure 3 and Table S3).

## 4. Discussion

Alternations of different single-gene crops (insecticides of different classes) across time of the same targeted pest are considered to delay the evolution of resistance based on computer simulation models and greenhouse cage experiments in certain conditions [17,29]. However, results from the present study indicate that the toxins used in a rotation fashion cannot delay resistance evolution more effectively than when used continuously. Contrary to the conditions in the models, the mortality caused by each toxin alone was, at most, 50%, resistance was not recessive, refuges were absent, and some cross-resistance occurred between the toxins. Results from our previous tests also demonstrated that pairs of toxins did not consistently delay the evolution of resistance relative to single toxins under the same conditions as above [15].

Cross-resistance may account for the present results, in that selection by either toxin promotes the evolution of resistance to the other two toxins. Previous studies have evaluated the cross-resistance between Cry1Ab, Cry1F, and Cry1Ie [15,24,25]. Moderate cross-resistance in *O. furnacalis* between Cry1Ab and Cry1F has been reported [25]. Selection with either Cry1Ab or Cry1F did not cause cross-resistance to Cry1Ie [24,25]. Amino acid sequence similarity between toxins of domain II is expected to influence cross-resistance when resistance is associated with reduced binding to midgut receptors [12]. The lack of cross-resistance between Cry1Ab or Cry1F and Cry1Ie may be associated with 44% and 40% amino acid sequence similarity of domain II, respectively [15]. 

The core to the success of the alternation and pyramid IRM strategies is that insect individuals resistant to one toxin are killed by the other; thus, no cross-resistance occurs between toxins [29,30]. However, the fact in the field often deviates substantially from those ideal conditions. Cross-resistance occurs when selection for resistance to a toxin causes resistance to a second toxin. An analysis of 80 cases involving 10 major pests to 7 sets of Bt toxins showed that cross-resistance between toxins used is pervasive [12]. A moderate to high level of cross-resistance (RR > 10) is expected to accelerate the evolution of resistance. By contrast, a lower level of cross-resistance is expected to accelerate the evolution of resistance only for insects that have inherently low susceptibility to the Bt toxins [12]. 

Insects that have evolved resistance to Cry1Ab, Cry1F, and Cry1Ie have been shown to develop polygenic inheritance in ACB [23,25,31]. In this study, evolution of resistance increased slowly to Cry1Ab vs. quickly to Cry1F and/or Cry1Ie, suggesting that resistance to either Cry1F or Cry1Ie, but not to Cry1Ab, may be governed by a set of closely linked loci. Quantitative trait locus (QTL) or unlinked genetic loci may confer resistance to Cry1Ab. This is similar to the results that resistance to Cry1F and Cry1Ab is conferred by tightly linked and unlinked genetic loci, respectively, in the ECB, a relative species of ACB [32,33]. In addition, any fitness costs associated with resistance will affect an insecticide alternation strategy [34,35]. In the present study, we did not assess the fitness costs associated with Bt resistance in all the strains of ACB, and it was, thus, not possible to accurately estimate what role does fitness cost plays in the evolution of resistance.

Given that the development of transgenic cultivars is a commercial exercise and developed by competing companies, it seems that farmers may plant crops in rotation patterns over different years. However, our selection experiment showed that multiple single-gene transgenic crops applied as rotation patterns do not slow the evolution of resistance to Cry1F or Cry1Ie. The lab selection experiment may not reflect the possible dynamics of resistance development in the field as the actual conditions are quite different from indoors. However, it is much easier/faster and can be used as a preliminary screening tool to identify “ideal” rotation regimes to be further verified in the greenhouse or open fields. The rotation of Bt crops in the open field is difficult to conduct because multiple generations are needed, and variables, such as climatic conditions, insect movement patterns, gene flow among different geographical populations, and natural enemies, may affect the results [36,37]. However, field studies are helpful in improving forecasts and promoting better resistance-management practices. 

## Data Availability

The data presented in this study are available on request from the corresponding author.

## Supplementary Materials

The following supporting information can be downloaded at: https://www.mdpi.com/article/10.3390/insects14010074/s1, Table S1: Evolution of resistance to Cry1Ab in *Ostrinia furnacalis* with different selection regimes, Table S2: Evolution of resistance to Cry1F in *Ostrinia furnacalis* with different selection regimes, Table S3: Evolution of resistance to Cry1Ie in *Ostrinia furnacalis* with different selection regimes.
