# Supplementary material for: Rotation of Multiple Single-Gene Transgenic Crops Did Not Slow the Evolution of Resistance to Cry1F or Cry1Ie in Ostrinia furnacalis
Source: Insects. 2023 Jan 12;14(1):74. doi: 10.3390/insects14010074 (PMC9866647; doi:10.3390/insects14010074)
Supplement: Supplementary file 1 [file insects-14-00074-s001.zip › Table S1.pdf]

Table S1 Evolution of resistance to Cry1Ab in *Ostrinia furnacalis* with different selection regimes

Selection regimes	Gen.	n	LC_{50} (95% FL) μg/g	RR (95% CI)	Slope ± SE	χ^2	df (χ^2)
Of-AbR	0	672	0.21(0.16 - 0.27)	1.07(0.83 - 1.38)	2.31 ± 0.36	14.9	12
	1	720	0.27(0.05 - 0.41)	1.36(0.75 - 2.48)	1.58 ± 0.45	14.7	13
	2	720	0.22(0.13 - 0.35)	1.12(0.73 - 1.73)	0.84 ± 0.09	15.9	13
	3	720	0.17(0.08 - 0.29)	0.85(0.51 - 1.43)	0.85 ± 0.11	18.4	13
	4	480	0.75(0.48 - 1.42)	3.86(2.33 - 6.40)	1.14 ± 0.26	2.3	8
	5	480	0.90(0.69 - 1.14)	4.66(3.49 - 6.22)	1.78 ± 0.19	3.4	8
	6	528	1.12(0.89 - 1.37)	5.76(4.45 - 7.45)	1.65 ± 0.14	4.5	9
	7	480	2.57(2.02 - 3.14)	13.24(10.16 - 17.24)	1.98 ± 0.19	5.3	8
	8	528	3.59(3.07-4.21))	18.51(14.92 - 22.96)	1.88 ± 0.14	5.6	9
	9	576	4.55(3.69-5.51)	23.44(18.28 - 30.04)	1.75 ± 0.15	6.7	10
	11	576	6.21(4.80 - 7.70)	32.02(24.27 - 42.25)	1.84 ± 0.17	3.5	10
	12	576	7.53(6.32 - 9.01)	38.83(30.82 - 48.89)	1.55 ± 0.11	6.5	10
	13	672	6.10(4.91 - 7.36)	31.37(24.45 - 40.25)	1.93 ± 0.23	8.3	12
	14	576	5.49(4.42 - 6.80)	28.24(22.62 - 35.26)	1.73 ± 0.12	5.5	10
Bi-alt.1	0	480	0.18(0.14 - 0.22)	0.92(0.69 - 1.21)	1.41 ± 0.16	6.3	8
	1	480	0.30(0.17 - 0.39)	1.53(1.14 - 2.05)	2.52 ± 0.41	11.6	8
	2	480	0.78(0.50 - 1.06)	4.11(2.85 - 5.93)	2.70 ± 0.79	7.1	8
	3	576	2.01(1.23 - 2.71)	10.36(7.08 - 15.17)	1.50 ± 0.33	5.3	10
	4	576	3.42(2.66 - 4.34)	17.62(13.25 - 23.44)	1.51 ± 0.18	3.7	10
	5	624	3.06(2.13 - 4.14)	15.77(11.01 - 22.59)	1.26 ± 0.13	1.3	11
	6	672	4.01(2.87 - 5.29)	20.63(14.71 - 28.95)	1.35 ± 0.14	7.6	12
	7	576	4.13(2.99 - 5.50)	21.31(15.21 - 29.84)	1.13 ± 0.11	5.3	10
	8	672	4.20(3.02 - 5.53)	21.62(15.43 - 30.30)	1.42 ± 0.13	5.9	12
	9	672	9.94(7.60 - 12.69)	51.24(38.14 - 68.83)	1.38 ± 0.13	4.9	12
	10	672	10.96(7.15 - 14.98)	56.38(38.25 - 83.09)	1.23 ± 0.17	5.6	12
	11	672	11.68(9.33 - 14.32)	60.23(46.49 - 78.03)	1.66 ± 0.15	4.3	12
	12	672	12.23(9.42 - 15.66)	62.94(46.86 - 84.53)	1.26 ± 0.12	5.1	12
	13	672	14.46(11.69 - 17.30)	74.56(58.46 - 95.08)	2.16 ± 0.22	9.5	12
	14	672	20.21(15.43 - 25.44)	103.96(77.80 - 138.90)	1.54 ± 0.16	5.1	12
Bi-alt.2	0	480	0.18(0.14 - 0.22)	0.92(0.69 - 1.21)	1.41 ± 0.16	6.3	8
	1	480	0.30(0.17 - 0.39)	1.53(1.14 - 2.05)	2.52 ± 0.41	11.6	8
	2	480	0.41(0.31 - 0.57)	2.11(1.64 - 2.72)	1.51 ± 0.14	12.2	8
	3	480	0.69(0.49 - 0.94)	3.57(2.50 - 5.09)	1.27 ± 0.17	6.1	8
	4	528	0.56(0.48 - 0.65)	2.89(2.34 - 3.55)	2.05 ± 0.15	2.0	9
	5	528	1.61(1.04 - 2.09)	8.28(5.80 - 11.83)	2.07 ± 0.35	8.7	9
	6	576	3.16(2.40 - 4.12)	16.31(12.0 - 22.16)	1.24 ± 0.12	4.0	10
	7	672	6.56(4.68 - 8.64)	33.84(24.21 - 47.32)	1.39 ± 0.17	4.2	12
	8	672	5.12(3.69 - 6.82)	26.41(18.78 - 37.14)	1.14 ± 0.10	7.9	12
	9	528	5.83(3.76 - 7.85)	30.06(20.56 - 43.95)	1.63 ± 0.25	6.3	9
	10	672	9.34(7.22 - 11.78)	48.13(36.19 - 64.02)	1.36 ± 0.12	5.2	12

	11	672	9.87(7.77- 12.11)	50.86(38.99 - 66.33)	1.70 ± 0.15	5.3	12
	12	672	17.41(14.33 - 21.53)	89.74(69.79 - 115.39)	1.34 ± 0.10	8.1	12
	13	768	20.95(15.59 - 26.53)	107.98(79.99 - 145.75)	1.51 ± 0.17	11.9	14
	14	672	26.18(20.25 - 32.59)	134.95(102.30 - 178.02)	1.66 ± 0.19	6.0	12
Tri-alt.1	0	480	0.18(0.14 - 0.22)	0.92(0.69 - 1.21)	1.41 ± 0.16	6.3	8
	1	480	0.30(0.17 - 0.39)	1.53(1.14 - 2.05)	2.52 ± 0.41	11.6	8
	2	480	0.71(0.51 - 0.98)	3.67(2.76 - 4.88)	1.82 ± 0.35	8.7	8
	3	432	0.93(0.69 - 1.21)	4.78(3.48 - 6.56)	1.52 ± 0.19	5.3	7
	4	576	1.40(0.53 - 1.99)	7.19(4.27 - 12.13)	1.59 ± 0.44	9.7	10
	5	528	0.99(0.74 - 1.27)	5.09(3.74 - 6.94)	1.38 ± 0.13	1.1	9
	6	624	1.33(0.99 - 1.71)	6.86(5.04 - 9.33)	1.36 ± 0.12	2.8	11
	7	576	7.82(6.02 - 9.87)	40.29(30.27 - 53.63)	1.53 ± 0.16	4.4	10
	8	624	4.87(3.51 - 6.48)	25.08(17.84 - 35.25)	1.21 ± 0.11	3.6	11
	9	672	4.12(3.17 - 5.21)	21.19(15.84 - 28.37)	1.23 ± 0.10	5.5	12
	10	624	5.0(4.01 - 6.14)	25.80(19.92 - 33.40)	1.52 ± 0.12	4.1	11
	11	672	9.32(6.85 - 12.07)	47.94(34.84 - 65.98)	1.44 ± 0.14	4.7	12
	12	672	13.02(10.19 - 17.14)	66.99(49.60 - 90.47)	1.0 ± 0.09	7.4	12
	13	672	15.06(10.63 - 20.10)	77.61(54.89 - 109.72)	1.24 ± 0.15	8.8	12
	14	624	11.71(9.69 - 14.26)	60.24(47.15 - 76.97)	1.37 ± 0.10	7.3	11
Tri-alt.2	0	480	0.18(0.14 - 0.22)	0.92(0.69 - 1.21)	1.41 ± 0.16	6.3	8
	1	480	0.30(0.17 - 0.39)	1.53(1.14-2.05)	2.52 ± 0.41	11.6	8
	2	480	0.71(0.51 - 0.98)	3.67(2.76-4.88)	1.82 ± 0.35	8.7	8
	3	432	0.93(0.69 - 1.21)	4.78(3.48-6.56)	1.52 ± 0.19	5.3	7
	4	624	1.35(1.11 - 1.63)	6.92(5.42-8.85)	1.36 ± 0.10	4.1	11
	5	576	0.88(0.66 - 1.13)	4.54(3.35-6.16)	1.25 ± 0.11	2.1	10
	6	624	3.61(2.45 - 4.99)	18.55(12.65-27.19)	1.12 ± 0.13	6.1	11
	7	672	7.35(5.34 - 9.46)	37.83(27.54-51.95)	1.54 ± 0.20	6.4	12
	8	528	3.60(2.63 - 4.71)	18.54(13.38-25.68)	1.23 ± 0.13	5.3	9
	9	624	5.43(4.50 - 6.57)	28.01(22.04-35.60)	1.38 ± 0.10	10.8	11
	10	576	7.74(5.72 - 9.71)	39.83(29.55-53.67)	1.89 ± 0.24	5.8	10
	11	672	12.98(10.19 - 16.23)	66.77(50.62-88.07)	1.45 ± 0.13	7.3	12
	12	672	20.69(11.64 - 29.07)	106.42(75.52-149.96)	1.70 ± 0.25	17.6	12
	13	672	20.91(15.10 - 26.47)	107.80(79.17-146.79)	1.89 ± 0.25	9.6	12
	14	672	22.09(17.80 - 26.56)	113.62(88.62 -145.68)	2.05 ± 0.21	7.2	12

Of-AbR, selected with Cry1Ab alone.

Bi-alt.1, selected with Cry1Ab - Cry1F in alternation.

Bi-alt.2, selected with Cry1Ab - Cry1Ie in alternation.

Tri-alt.1, selected with Cry1Ab - Cry1F - Cry1Ie in alternation.

Tri-alt.2, selected with Cry1Ab - Cry1F - Cry1Ie - Cry1F in alternation.
