# Supplementary material for: Rotation of Multiple Single-Gene Transgenic Crops Did Not Slow the Evolution of Resistance to Cry1F or Cry1Ie in Ostrinia furnacalis
Source: Insects. 2023 Jan 12;14(1):74. doi: 10.3390/insects14010074 (PMC9866647; doi:10.3390/insects14010074)
Supplement: Supplementary file 1 [file insects-14-00074-s001.zip › Table S2.pdf]

Table S2 Evolution of resistance to Cry1F in *Ostrinia furnacalis* with different selection regimes

Selection regimes	Gen.	n	LC_{50} (95% FL) μg/g	RR (95% CI)	Slope ± SE	χ^2	df (χ^2)
Of-FR	0	720	0.54(0.40 - 0.69)	0.68(0.52 - 0.89)	2.47 ± 0.31	19.3	13
	1	720	0.80(0.64 - 0.97)	1.01(0.77 - 1.33)	2.29 ± 0.27	12.6	13
	3	720	1.07(0.89 - 1.28)	1.35(1.04 - 1.75)	2.38 ± 0.24	5.3	13
	4	480	2.09(0.86 - 3.02)	2.63(1.79 - 3.88)	1.71 ± 0.39	10.7	8
	5	528	48.20(38.63 - 59.09)	58.96(44.65 - 77.84)	1.82 ± 0.18	3.3	9
	6	672	441.96(365.56 - 604.33)	556.78(418.95 - 739.94)	2.51 ± 0.61	9.7	12
	7	96	> 476	> 600			
	9	96	> 1030	> 1300			
	12	96	> 1000	> 1250			
	14	96	> 1000	> 1250			
Bi-alt.1	0	576	0.54(0.27 - 0.84)	0.68(0.47 - 0.99)	1.39 ± 0.18	19.0	10
	1	480	0.80(0.37 - 1.34)	1.01(0.71 - 1.45)	1.40 ± 0.20	18.4	8
	2	576	0.87(0.69 - 1.08)	1.10(0.82 - 1.47)	1.61 ± 0.15	7.7	10
	3	528	5.22(4.20 - 6.34)	6.58(5.0 - 8.65)	2.03 ± 0.22	4.5	9
	4	672	50.71(38.74 - 68.65)	63.88(45.56 - 89.58)	1.20 ± 0.13	4.4	12
	5	672	177.06(143.8 - 215.7)	223.06(171.24 - 290.55)	2.27 ± 0.43	7.6	12
	6	576	> 300	> 380			
	7	96	> 476	> 600			
	11	96	> 1010	> 1250			
	14	96	> 1000	> 1250			
Tri-alt.1	0	576	0.54(0.27 - 0.84)	0.68(0.47 - 0.99)	1.39 ± 0.18	19.0	10
	1	480	0.80(0.37 - 1.34)	1.01(0.71 - 1.45)	1.40 ± 0.20	18.4	8
	2	624	1.03(0.77 - 1.30)	1.29(0.94 - 1.77)	1.57 ± 0.15	4.5	11
	3	480	4.05(2.98 - 5.03)	5.11(3.75 - 6.95)	2.15 ± 0.35	3.8	8
	4	576	48.8(39.70 - 58.61)	61.48(47.21 - 80.06)	2.21 ± 0.30	6.9	10
	5	672	180.25(141.3 - 241.6)	227.07(164.50 - 313.45)	1.39 ± 0.15	6.3	12
	6	96	> 476	> 600			
	7	96	> 476	> 600			
	10	96	> 1000	> 1250			
	12	96	> 1000	> 1250			
Tri-alt.2	0	576	0.54(0.27 - 0.84)	0.68(0.47 - 0.99)	1.39 ± 0.18	19.0	10
	1	480	0.80(0.37 - 1.34)	1.01(0.71 - 1.45)	1.40 ± 0.20	18.4	8
	2	624	1.03(0.77 - 1.30)	1.29(0.94 - 1.77)	1.57 ± 0.15	4.5	11
	3	480	4.05(2.98 - 5.03)	5.11(3.75 - 6.95)	2.15 ± 0.35	3.8	8
	4	672	68.74(56.15 - 83.70)	86.60(66.11 - 113.45)	1.72 ± 0.17	3.8	12
	5	672	158.39(124.53 - 196.40)	199.54(149.21 - 266.84)	1.52 ± 0.17	3.3	12
	6	672	> 476	> 600			
	7	96	> 476	> 600			
	10	96	> 1000	> 1250			
	14	96	> 1000	> 1250			

Of-FR, selected with Cry1F alone.

Bi-alt.1, selected with Cry1Ab - Cry1F in alternation.

Tri-alt.1, selected with Cry1Ab - Cry1F - Cry1Ie in alternation.

Tri-alt.2, selected with Cry1Ab - Cry1F - Cry1Ie - Cry1F in alternation.
