# Supplementary material for: Rotation of Multiple Single-Gene Transgenic Crops Did Not Slow the Evolution of Resistance to Cry1F or Cry1Ie in Ostrinia furnacalis
Source: Insects. 2023 Jan 12;14(1):74. doi: 10.3390/insects14010074 (PMC9866647; doi:10.3390/insects14010074)
Supplement: Supplementary file 1 [file insects-14-00074-s001.zip › Table S3.pdf]

**Table S3** Evolution of resistance to Cry1Ie in *Ostrinia furnacalis* with different selection regimes

| Selection regimes | Gen. | n | $LC_{50}$ (95% FL) μg/g | RR (95% CI) | Slope ± SE | $\chi^2$ | df ($\chi^2$) |
|---|---|---|---|---|---|---|---|
| *Of*-IeR | 0 | 576 | 1.25(1.07 - 1.42) | 0.52(0.43 - 0.62) | 5.60 ± 0.71 | 15.0 | 10 |
| | 1 | 576 | 1.55(1.32 - 1.75) | 0.64(0.53 - 0.77) | 4.37 ± 0.66 | 10.5 | 10 |
| | 2 | 576 | 1.84(1.61 - 2.05) | 0.76(0.63 - 0.91) | 5.05 ± 0.67 | 10.4 | 10 |
| | 3 | 480 | 2.19(0.77 - 4.14) | 0.90(0.56 - 1.47) | 1.11 ± 0.15 | 15.1 | 8 |
| | 4 | 480 | 7.39(5.72 - 9.32) | 3.04(2.29 - 4.04) | 1.66 ± 0.20 | 6.8 | 8 |
| | 6 | 576 | 269.91(198.19 - 396.59) | 111.11(77.33 - 159.64) | 1.36 ± 0.25 | 8.6 | 10 |
| | 7 | 672 | > 952 | > 400 | | | |
| | 9 | 96 | > 1935 | > 800 | | | |
| | 12 | 96 | > 2480 | > 1000 | | | |
| | 14 | 96 | > 2047 | > 840 | | | |
| Bi-alt.2 | 0 | 480 | 2.45(1.73 - 3.35) | 1.01(0.74 - 1.37) | 1.36 ± 0.15 | 8.8 | 8 |
| | 1 | 480 | 3.42(2.28 - 5.12) | 1.41(1.0 - 1.98) | 1.14 ± 0.14 | 9.5 | 8 |
| | 2 | 576 | 6.92(4.87 - 10.61) | 2.85(2.19 - 3.70) | 1.36 ± 0.11 | 24.3 | 10 |
| | 3 | 384 | 3.39(2.74 - 4.10) | 1.39(1.09 - 1.79) | 2.11 ± 0.24 | 1.1 | 6 |
| | 4 | 576 | 28.72(22.75 - 37.50) | 11.82(8.82 - 15.85) | 1.14 ± 0.10 | 4.1 | 10 |
| | 5 | 576 | 115.53(85.98 - 157.85) | 47.56(33.97 - 66.58) | 1.27 ± 0.16 | 7.9 | 10 |
| | 6 | 672 | > 625 | > 250 | | | |
| | 7 | 96 | > 952 | > 400 | | | |
| | 10 | 96 | > 1820 | > 750 | | | |
| | 14 | 96 | > 2780 | > 1100 | | | |
| Tri-alt.1 | 0 | 480 | 2.45(1.73 - 3.35) | 1.01(0.74 - 1.37) | 1.36 ± 0.15 | 8.8 | 8 |
| | 1 | 480 | 3.42(2.28 - 5.12) | 1.41(1.0 - 1.98) | 1.14 ± 0.14 | 9.5 | 8 |
| | 2 | 480 | 5.0(3.62 - 7.29) | 2.06(1.55 - 2.74) | 1.48 ± 0.16 | 11.5 | 8 |
| | 3 | 480 | 7.31(5.46 - 8.94) | 3.01(2.27 - 3.98) | 2.38 ± 0.37 | 7.1 | 8 |
| | 4 | 576 | 36.07(25.57 - 46.16) | 14.85(10.80 - 20.42) | 1.72 ± 0.30 | 4.5 | 10 |
| | 5 | 672 | 324.2(231.9 - 476.7) | 133.46(90.84 - 196.07) | 1.09 ± 0.15 | 5.4 | 12 |
| | 6 | 672 | > 952 | > 400 | | | |
| | 7 | 96 | > 952 | > 400 | | | |
| | 10 | 96 | > 2170 | > 900 | | | |
| | 14 | 96 | > 2920 | > 1200 | | | |
| Tri-alt.2 | 0 | 480 | 2.45(1.73 - 3.35) | 1.01(0.74 - 1.37) | 1.36 ± 0.15 | 8.8 | 8 |
| | 1 | 480 | 3.42(2.28 - 5.12) | 1.41(1.0 - 1.98) | 1.14 ± 0.14 | 9.5 | 8 |
| | 2 | 480 | 5.0(3.62 - 7.29) | 2.06(1.55 - 2.74) | 1.48 ± 0.16 | 11.5 | 8 |
| | 3 | 480 | 7.31(5.46 - 8.94) | 3.01(2.27 - 3.98) | 2.38 ± 0.37 | 7.1 | 8 |
| | 4 | 576 | 45.65(36.23 - 60.06) | 18.79(13.99 - 25.25) | 1.25 ± 0.11 | 5.7 | 10 |
| | 5 | 576 | 354.4(283.2 - 464.6) | 145.91(110.39 - 192.85) | 1.92 ± 0.36 | 7.1 | 10 |
| | 6 | 672 | > 952 | > 400 | | | |
| | 7 | 96 | > 952 | > 400 | | | |
| | 10 | 96 | > 2010 | > 800 | | | |
| | 14 | 96 | > 3680 | > 1500 | | | |

*Of*-IeR, selected with Cry1Ie alone.

Bi-alt.2, selected with Cry1Ab - Cry1Ie in alternation.

Tri-alt.1, selected with Cry1Ab - Cry1F - Cry1Ie in alternation.

Tri-alt.2, selected with Cry1Ab - Cry1F - Cry1Ie - Cry1F in alternation.
